# Effects of exogenous nerve growth factor on the expression of BMP-9 and VEGF in the healing of rabbit mandible fracture with local nerve injury

**DOI:** 10.1186/s13018-021-02220-z

**Published:** 2021-01-21

**Authors:** Sen Yang, Jiao Cheng, Cheng Man, Lian Jiang, Guogeng Long, Wenjun Zhao, Dexin Zheng

**Affiliations:** Oral Maxillofacial Trauma and Orthognathic Surgery, Affiliated Stomatological Hospital of Zunyi Medical University, Zunyi, Guizhou China

**Keywords:** Mandibular fractures, Nerve growth factor, Vascular endothelial growth factor, Bone morphogenic protein-9, Fracture healing, Rabbits

## Abstract

**Background:**

Mandibular fracture healing is a complex process involving nerves and growth factors. Nerve growth factor (NGF) not only facilitates the maintenance of sympathetic neurite growth but also stimulates other growth factors that can promote the essential osteogenesis and angiogenesis for fracture healing. Therefore, it is necessary to analyze the combined effects of NGF, bone morphogenic protein-9 (BMP-9), and vascular endothelial growth factor (VEGF) to accelerate the healing of mandible fractures.

**Methods:**

The models of mandible fracture with local nerve injury established in 48 rabbits were randomly divided into nerve growth factor group (NGF group), gelatin sponge group (GS group), blank group, and intact group. The recovery of nerve reflex was assessed by observing the number of rabbits with lower lip responses to acupuncture. The fracture healing was observed with visual and CBCT, and then callus tissues from the mandibular fracture area were collected for hematoxylin and eosin (HE) staining observation, and the expression of BMP-9 and VEGF in callus at different stages was detected by quantitative real-time PCR (qRT-PCR).

**Results:**

Needling reaction in the lower lip showed the number of animals with nerve reflex recovery was significantly higher in the NGF group than that in the GS and blank groups at the 2nd and 4th weeks after the operation. The combined results of macroscopic observation, CBCT examination, and histological analysis showed that a large number of osteoblasts and some vascular endothelial cells were found around the trabecular bone in the NGF group and the amount of callus formation and reconstruction was better than that in the GS group at the 2nd week after the operation. The qRT-PCR results indicated that the expression levels of BMP-9 and VEGF in the four groups reached the highest values at the 2nd week, while the expression levels of both in the NGF group were significantly higher than that in the GS group.

**Conclusion:**

The exogenous NGF could accelerate the healing of mandible fractures. This work will provide a new foundation and theoretical basis for clarifying the mechanism of fracture healing, thereby promoting fracture healing and reducing the disability rate of patients.

**Supplementary Information:**

The online version contains supplementary material available at 10.1186/s13018-021-02220-z.

## Background

With nearly 29% of all maxillofacial fractures with a range of 11–36%, mandibular fracture remains one of the most common facial fractures [1, 2]. Many studies have reported that mandibular fracture patterns were usually from developing countries [3–5], and vehicular accidents have been described as the most frequent cause of all etiology factors, followed by assaults, falls, and sports [6]. As the only movable bone of the skull, the mandible is connected to the temporal bone by the temporomandibular joint and is rich with the inferior alveolar vessels and nerve bundles. The special nature of the mandible anatomy determines that mandibular fractures are often accompanied by damage of the nerve bundles. Healing of fracture is an instinctive physiological response that requires coordination and refers to the regeneration and repair reactions of the local bone tissue at the fracture site controlled by nerve regulation and humoral regulation [7–10]. Explicating the molecular mechanism of fracture healing and then finding proper methods to treat mandibular fracture become more important.

Many studies have investigated the effect of regulating factors including cell proliferation and differentiation, matrix synthesis, and calcification in the process of fracture healing [8–10]. These factors are jointly affected by nerve regulation and humoral regulation, such as nerve growth factor (NGF), insulin-like growth factor 1 (IGF1), bone morphogenic protein-2 (BMP-2), and so on [11–14]. NGF, as a neurotrophic substance, not only plays an important role in maintaining the growth of sympathetic neurites [15] and increasing the activity of enzymes associated with catecholamine synthesis [16], but also is participated in accelerating the rate of fracture healing with brain injury and skeleton development [11, 17–19]. Some studies showed that NGF stimulated sympathetic neurite growth and influenced neurite extension after fracture compared with controls [20–23]. It has been reported that NGF could promote the proliferation and differentiation of bone cells and inhibit the formation of osteoclasts through endocrine factors such as other neuropeptides and hormones, thereby promoting fracture healing [24].

Belonging to the transforming growth factor-β (TGF-β) superfamily, more than 14 bone morphogenic proteins (BMPs) have been identified to induce ectopic bone growth and cartilage formation [25, 26]. Bone morphogenic protein-9 (BMP-9), a secreted protein [27], is known as growth differentiation factor 2 (GDF2) and is expressed in the liver [28]. BMP-9 has the most potent bone-forming capability and maybe participates in a variety of physiological and pathological functions such as osteogenesis, chondrogenesis, angiogenesis, and tumorigenesis via a complicated network of signaling pathways [29–31]. Osteogenesis and angiogenesis are essential for physiological bone formation, growth, and fracture healing. As a signal protein produced by cells, vascular endothelial growth factor (VEGF) is a powerful regulator of angiogenesis and vasculogenesis [32]. Skeleton is a highly vascularized tissue that relies on blood vessels and VEGF promotes the regeneration of blood vessels as an angiogenic factor, which is as important as in osteogenesis [33]. However, a single growth factor has a limited degree of influence on bone regeneration, and the concentration of cytokines and growth factors can accelerate the process of fracture repair [34].

To analyze the combination of NGF, BMP-9, and VEGF to accelerate the healing rate of mandible fracture, the rabbit models of mandible fracture with local nerve injury were constructed in this study. This study aims to investigate the regulating effects of exogenous nerve growth factor on the expression of BMP-9 and VEGF, and to further elucidate the possible mechanism of promoting fracture healing through exogenous NGF.

## Materials and methods

### Animals

Forty-eight skeletally-mature, male or female New Zealand white rabbits, weighing 2.5–3.0 kg (mean 2.7 ± 0.2), were included in the study. The animals were transferred to the experimental animal center of Zunyi Medical University at least 1 week before the surgery and kept in separate cages to help them adapt to the new environment as well as to ensure their health. This study was performed by the regulations of the Animal Management Regulations and Administrative Measures on Experimental Animal and approved by the medical ethics committee of Zunyi Medical University (Approval No. 2018. 246).

### Establishment of rabbit fracture model

Forty-eight healthy rabbits were used to establish fracture models by the following surgery under aseptic conditions. All rabbits were randomly selected and placed in the supine position, and their submandibular region was prepared individually. They were anesthetized with an ear vein injection of 3% pelltobarbitalum natricum (1 ml/kg body weight), and 2% lidocaine (2 ml) was then injected for intensive local anesthesia. After submandibular incision and dissection of the periosteum, both sides of the mandible anterior to the masseter muscle were exposed by blunt and sharp dissection, and the neurovascular bundle of the mandible of 36 rabbits was transected. Then, incomplete fractures (about 5 mm × 2 mm) were made in front of the mental foramen of the mandible through the buccal and lingual using diamond burs, and the zone was fully rinsed and cooled by physiological saline at the same time. All fracture models need no reduction and fixation.

### Experimental groups

Forty-eight New Zealand white rabbits were randomly assigned to the nerve growth factor group (NGF group), gelatin sponge group (GS group), blank group, and intact group with 12 rabbits in each group. In the NGF group, the mental neurovascular bundle was cut off and implanted l ml nerve growth factor (10 μg/ml) with gelatin sponge as the carrier. The mental neurovascular bundle was cut off and implanted with normal saline equivalent to NGF with gelatin sponge as the carrier in the GS group. The mental neurovascular bundle was cut off without implanting material serving as the blank group. The intact group retained the neurovascular bundle intact and no material was implanted.

### Postoperative care

After the rabbits were fully awakened from the operation, they were returned to separate cages, and the lower lip response to acupuncture was performed on the same day. Penicillin (0.4 million units) was administered intramuscularly to each rabbit twice a day for 3 days. Before the stitches were removed on the 7th day, the wound was cleaned and the healing status was observed every day.

### Observation of lower lip reaction to acupuncture

The mental nerve innervated the skin of the lower lip area in front of one side of the chin foramen. If the lower lip did not respond to acupuncture on the first day after the operation, the nerve fracture models were successfully established. Besides, the recovery of nerve reflex was assessed by observing the number of rabbits with lower lip responses to acupuncture at the 2nd, 4th, 6th, and 8th weeks after the operation.

### Visual observation and CBCT examination of the fracture zone

At the 2nd, 4th, 6th, and 8th weeks after the operation, three animals in each group were sacrificed and fracture healing was determined by observing and comparing the size of the bone gaps and the clarity of the osteotomy line using cone-beam computed tomography (CBCT) systems. The operated mandible of each executed rabbit was dissected subperiosteally to visually observe the formation of healing tissue at the fracture site as well as the amount of healing tissue formation and degree of fusion. The healing tissues were immediately collected for subsequent hematoxylin and eosin (HE) staining observation and qRT-PCR analysis.

### Histological observation

Callus tissues from the mandibular fracture area were collected using rongeur, rinsed in physiological saline, and then immediately put into 10% neutral buffered formalin fixative for 48 h. Subsequently, the samples were rinsed in running tap water for 10 min and incubated with 10% ethylenediaminetetraacetic acid (EDTA) (pH = 7.2). The decalcifying solution was changed every 3 days until the decalcification was completed. The decalcification process was finished when the specimen was easily penetrated by a needle without any force. Next, samples were washed in 0.01 M phosphate-buffered saline (PBS) for 10 min and then followed by routine dehydration and paraffin embedding. The paraffin wrapped tissues were cut into 4 μm sections using a Leica microtome (Leica, Germany). The tissue sections were soaked by xylene solution two times with 20 min to dewax the sections, and then soaked in 100%, 95%, 80%, and 75% alcohol for 5 min, and finally rinsed in running tap water.

After deparaffinization and rehydration, the sections were stained with hematoxylin dye for five minutes and then rinsed in running tap water. Soak the sections with 100%, 95%, 80%, and 75% alcohol to dehydrate the sections. The sections were soaked in xylene until the sections were clear, and then the tablet was sealed. Finally, the pathological changes of callus tissue between all groups were observed and photographed using an Olympus BX53 fluorescence microscope (Tokyo, Japan) by the following criteria: (1) the distribution of bone matrix collagen; (2) the distribution and number of osteoblasts; (3) the distribution and number of vascular endothelial cells; (4) the arrangement of trabecular.

### RNA extraction and quantitative real-time PCR

To determine whether exogenous NGF could improve the expression of BMP-9 and VEGF during mandibular fracture by detecting the expression levels of BMP-9 mRNA and VEGF mRNA in four groups of healing tissues at the 2nd, 4th, 6th, and 8th weeks after the operation. For RNA extraction, callus tissue in the mandibular fracture area was collected using rongeur and immediately frozen in liquid nitrogen. The frozen tissue was ground to a fine powder in liquid nitrogen using a freezer mill (Bone Mill; SPEX CertiPrep, Metuchen, NJ, USA). Total RNA was extracted using the RNeasy Mini Kit (Qiagen, Hilden, Germany), according to the manufacturer’s instruction. The quantity, degradation, and contamination of total RNA were assessed using a NanoDrop 2000 spectrophotometer (Thermo Scientific, Waltham, MA, USA) and 1% agarose gel electrophoresis, respectively.

RNAs were reverse-transcribed by oligo (dT) primer using the ThermoScriptTM RT–PCR system (Invitrogen, Carlsbad, CA, USA). QRT-PCR analysis was carried out using an ABI PRISM7300 Fast Real-Time PCR System (Applied Biosystems, Foster City, CA, USA). The published sequences of BMP-9, VEGF, and glyceraldehyde-3-phosphate dehydrogenase (GAPDH) were obtained from GeneBank, and these oligonucleotide primers for the rabbit-specific genes were designed using the Primer Express Software (Applied Biosystems), as shown in Table 1. GAPDH, a constitutively expressed housekeeping gene, was used as a control gene, and all gene expression data was calibrated to those for GAPDH. Gene expression quantitation was calculated with the comparative cross-threshold (Ct) method. The difference between the average Ct value of the gene of target and the GAPDH was expressed as (ΔCt), and ∆∆Ct equals the difference between the ΔCt and the Ct value of the calibrator sample. The 2^−ΔΔCt^ gave the relative quantitation value of gene expression.

**Table 1:** Sequence of primers

Gene	Genebank no.	Forward prime (5′-3′)	Reverse prime (5′-3′)
BMP-9	XM_017339607	ACCCTGGTGCATCTCAAGTT	GTAGAGGATGGAGATGGGGC
VEGF	XM_017345155	AACGAACGTACTTGCAGATGT	GCTCACGCAGTCTCCTCTTC
GAPDH	NM_001082253	AGAGCACCAGAGGAGGACGA	TGGGATGGAAACTGTGAAGAGG

*BMP-9* bone morphogenetic protein-9, *VEGF* vascular endothelial growth factor, *GAPDH* glyceraldehyde-3-phosphate dehydrogenase

### Statistical analysis

The descriptive values for BMP-9 and VEGF in different periods in the NGF group, GS group, blank group, and intact group were presented in mean and standard deviations. The statistical analysis was analyzed with SPSS 20.0, (IBM, Armonk, NY, USA). Statistical differences among groups were detected by one-way ANOVA followed by Bonferroni’s multiple comparison test. A *p* value of < 0.05 was considered statistically significant.

## Results

### Model establishment and evaluation

**Fig. 1:**
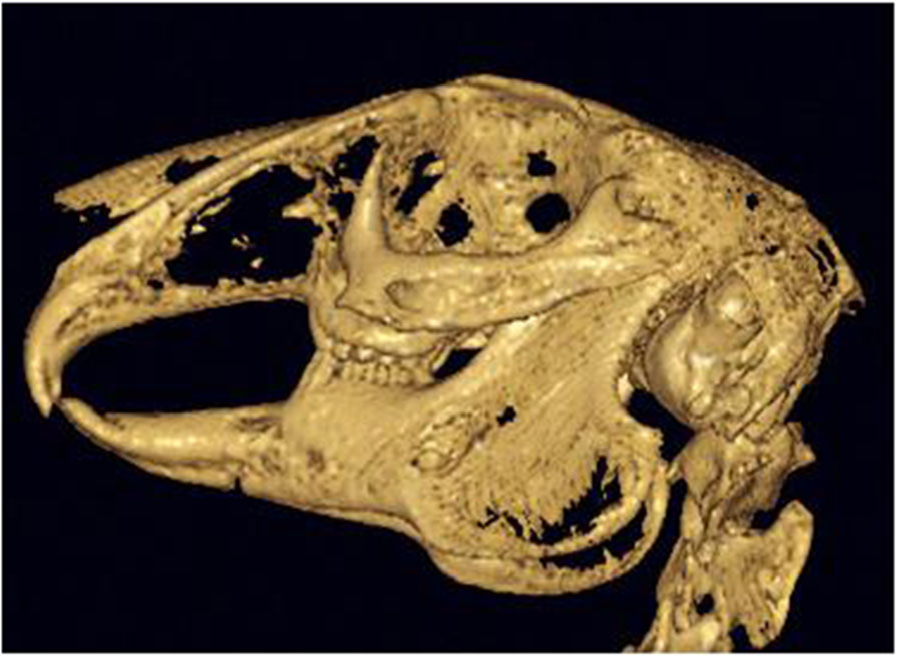
The result of CBCT examination after surgery

A total of 48 New Zealand white rabbits were included in this study. CBCT examination showed that incomplete fractures of the mandible were prepared successfully in all models (Fig. 1). The incisions healed well after the operation, without obvious swelling and bleeding, infection, or death. The mental neurovascular bundle was successfully severed in the NGF group, GS group, and blank group, and remained intact in the intact group.

### Acupuncture reaction on the lower lip

Acupuncture was performed on the lower lip on the first day after the operation. The results showed that there was a contraction reaction of the lower lip in the intact group, but no reaction in the NGF, GS, and blank group, which proved that the nerve dissection operation was successful (Table 2). At the 2nd and 4th weeks after the operation, the number of animals with nerve reflex recovery was higher in the NGF group (3 and 9, respectively) than that in the GS group (0 and 7, respectively) and the blank group (0 and 6, respectively). The results of acupuncture reaction at the 6th and 8th weeks after the operation indicated that the number of animals with nerve reflex recovery was the same in each group (Table 2).

**Table 2:** Needling reaction in lower lip (number of responding animals/number of remaining animals)

Group	1 day	2 weeks	4 weeks	6 weeks	8 weeks
Intact	12/12	12/12	9/9	6/6	3/3
NGF	0/12	3/12	9/9	6/6	3/3
GS	0/12	0/12	7/9	6/6	3/3
Blank	0/12	0/12	6/9	6/6	3/3

### Radiographic and histological appearance

#### 1 day after the operation

**Fig. 2:**
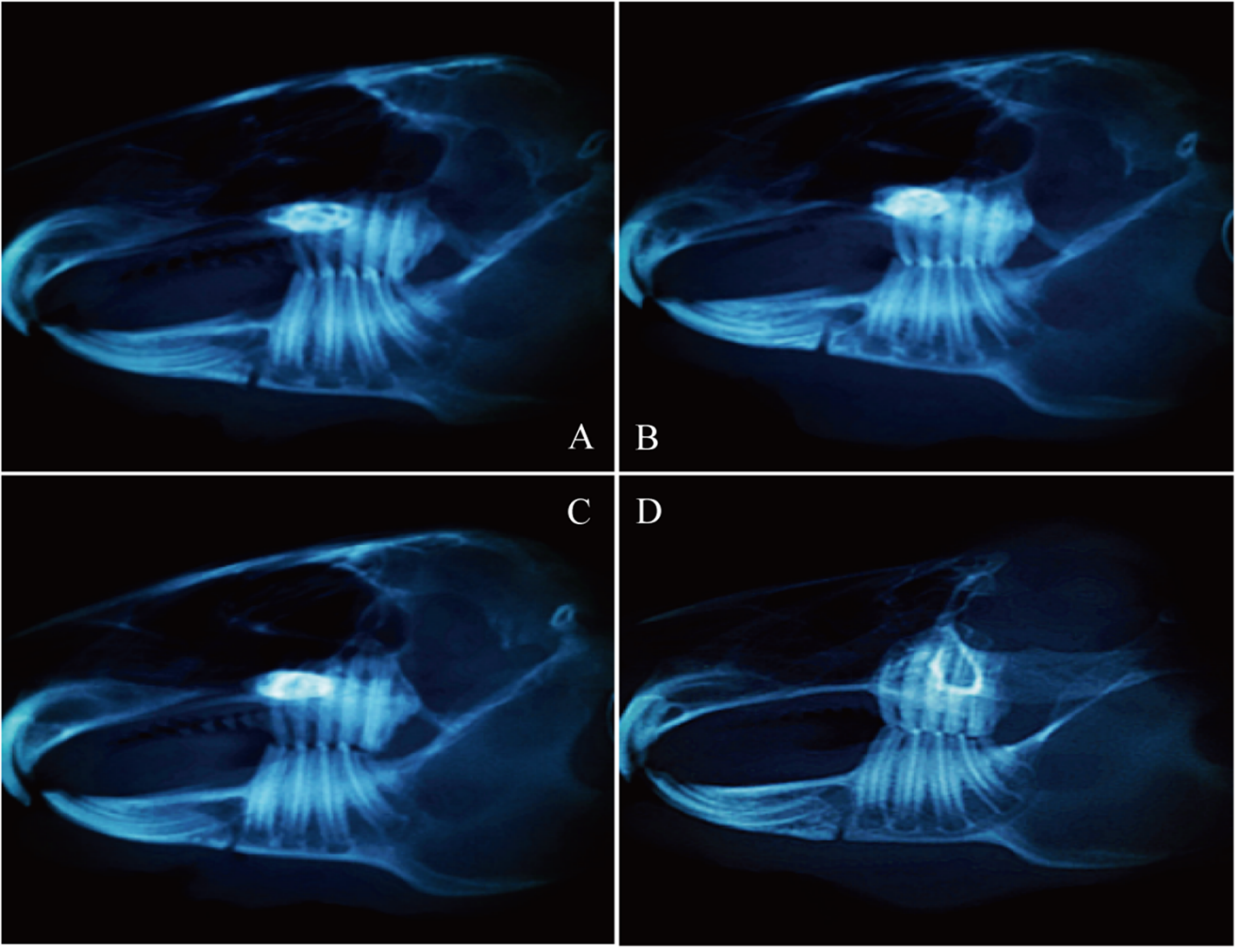
CBCT results on the first day after surgery. **a** Intact group. **b** NGF group. **c** GS group. **d** Blank group

The results of CBCT examination showed that the fracture situation of each group was roughly the same, with clear bone incision lines and obvious bone gap (Fig. 2).

#### 2 weeks after the operation

**Fig. 3:**
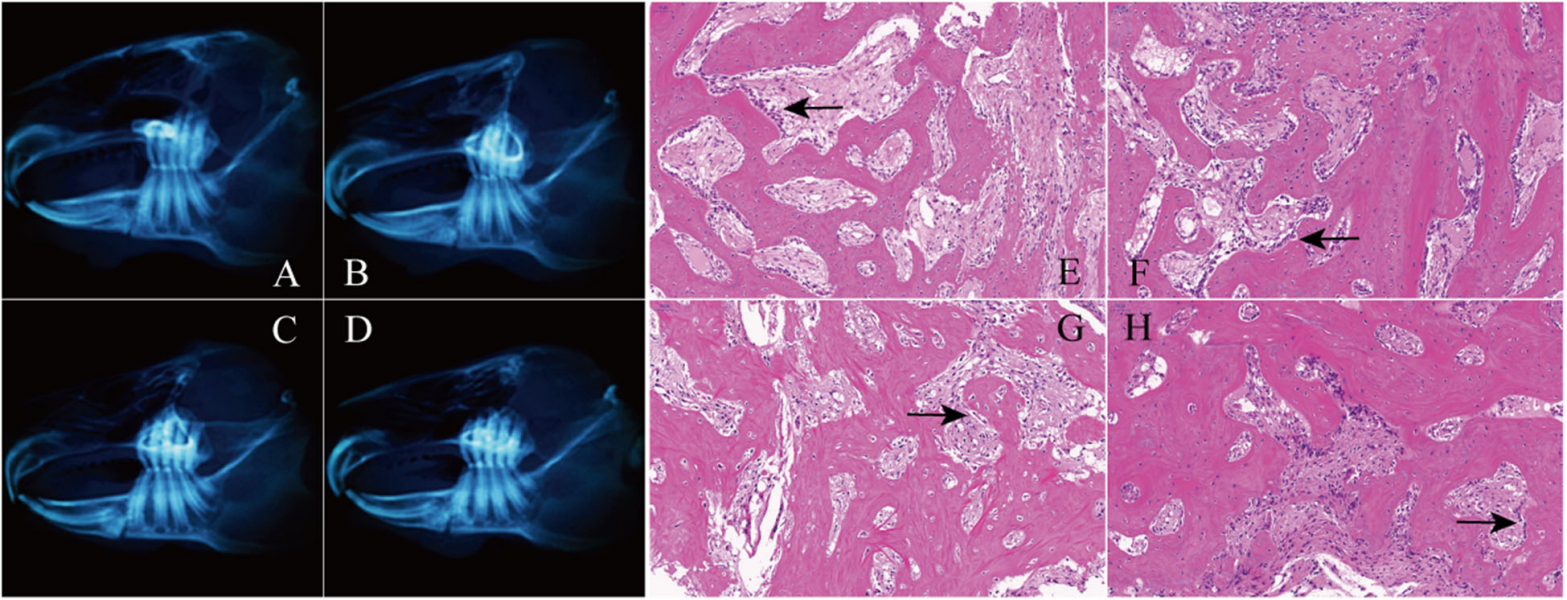
CBCT results and histological analysis at the 2nd week after surgery. **a**–**d** The CBCT results of the intact group, NGF group, GS group, and the blank group were shown. **e**–**h** Histological analysis of the intact group, NGF group, GS group, and the blank group was detected

Macroscopic observation and CBCT examination demonstrated that callus was formed in the fracture area of each group, and the osteotomy line was clear (Fig. 3a–d). The amount of callus formation in the intact group and NGF group was more than that in the GS group and blank group, but the osteotomy line was relatively fuzzy compared with the GS group and blank group. In the NGF group, there was adhesion at the broken ends of nerve vessels (Fig. 3b), but there was no obvious change in the broken neurovascular bundles between the GS group and the blank group (Fig. 3c, d). Histological analysis showed that compared with GS group and blank group (Fig. 3g, h), the distribution of collagen in the bone matrix of NGF group and intact group was more uniform, the process of callus reconstruction was more obvious, and a large number of osteoblasts and some vascular endothelial cells were found around the trabecular bone (Fig. 3e, f). However, the arrangement of trabeculae was irregular in all four groups at the point after the operation.

#### 4 weeks after the operation

**Fig. 4:**
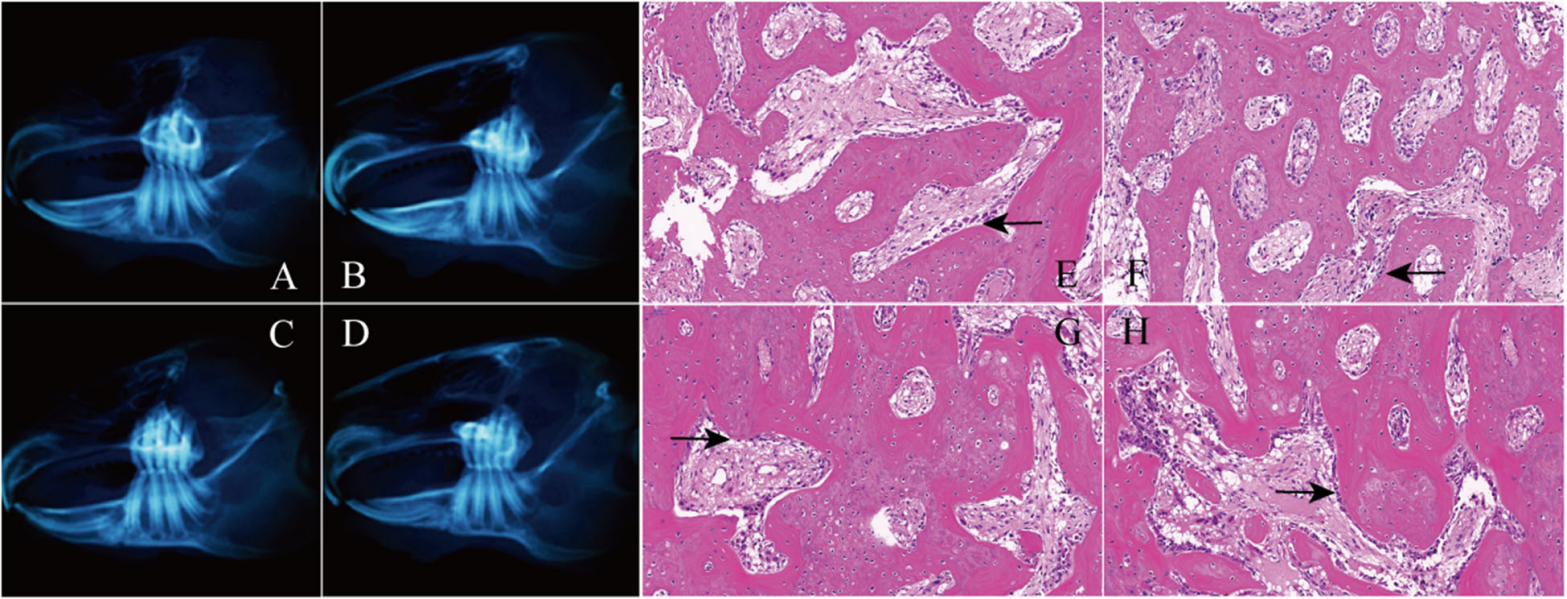
CBCT results and histological analysis at the 4th week after surgery. **a**–**d** The CBCT results of the intact group, NGF group, GS group, and the blank group were shown. **e**–**h** Histological analysis of the intact group, NGF group, GS group, and the blank group was detected

Macroscopic observation and CBCT examination indicated that the hyperplasia of the callus was obvious in all the four groups, and the bone gap was smaller than that at the 2nd week (Fig. 4a–d). Compared with the GS group and blank group, the osteotomy line in the fracture area became blurred in the NGF group and intact group, the amount of callus formation and the degree of fusion were better, and the bone gap became less obvious (Fig. 4a, b). The histological analysis showed that the trabecular arrangement became more regular in the NGF group and intact group, the process of callus reconstruction remained obvious, and a large number of osteoblasts were still visible around the trabecular bone (Fig. 4e, f). In the GS group and blank group, the arrangement of local bone trabeculae was still irregular, the distribution of collagen in the bone matrix became more uniform than before, the reconstruction process of osteocytes and callus was more obvious, and more osteoblasts began to appear around the trabeculae (Fig. 4g, h).

#### 6 weeks after the operation

**Fig. 5:**
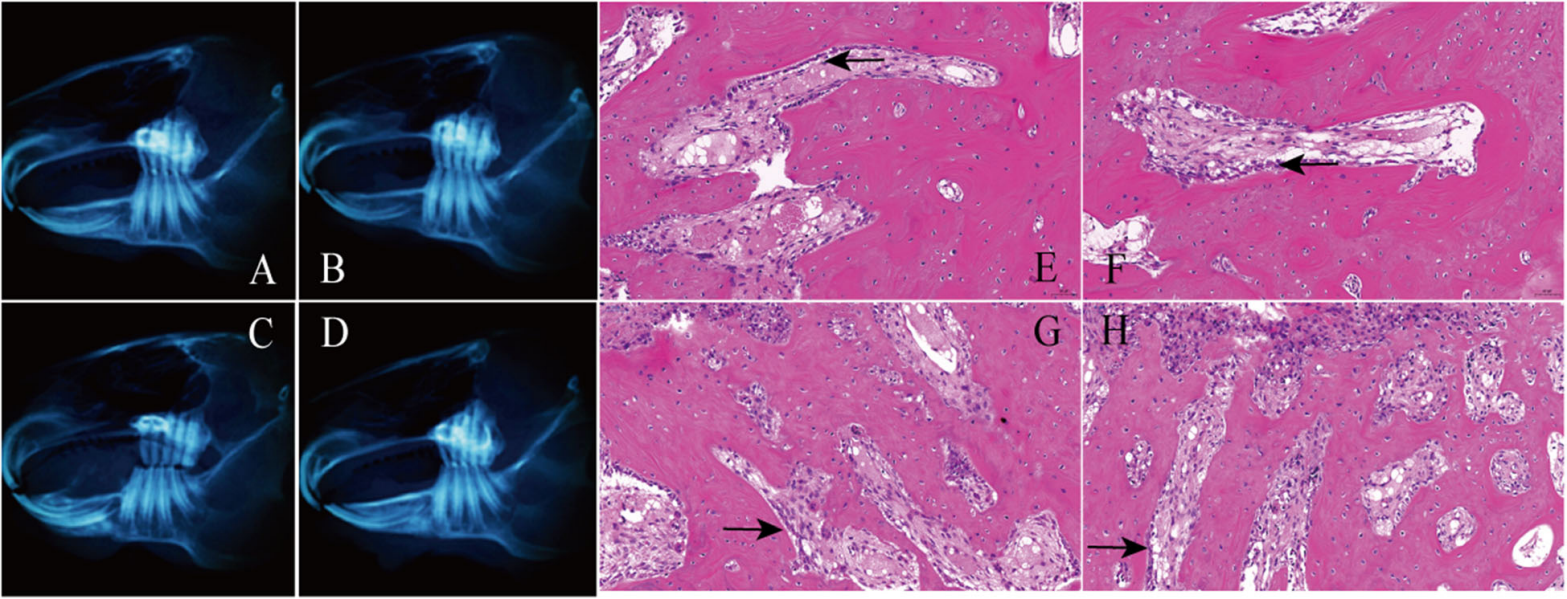
CBCT results and histological analysis at the 6th week after surgery. **a**–**d** The CBCT results of the intact group, NGF group, GS group, and blank group were shown. **e**–**h** Histological analysis of the intact group, NGF group, GS group, and the blank group was detected

Macroscopic observation and CBCT examination determined that the callus at both ends of the osteotomy line in the NGF group and the intact group had fused and calcified, and the fracture line disappeared (Fig. 5a, b). Fusion calcification appeared in the callus at both ends of the osteotomy line in the GS group and blank group, and there were still ambiguous fracture lines. The regeneration of the ruptured neurovascular bundle was also basically completed in the GS group and blank group (Fig. 5c, d). Histological analysis showed that the trabecular in the four groups were relatively regular in the arrangement, the distribution of bone matrix collagen tended to be uniform, and there were more columnar osteoblasts around the trabeculae. However, the process of callus reconstruction was not obvious in the NGF group and intact group, while remained active in the GS group and blank group (Fig. 5e–h).

#### 8 weeks after the operation

**Fig. 6:**
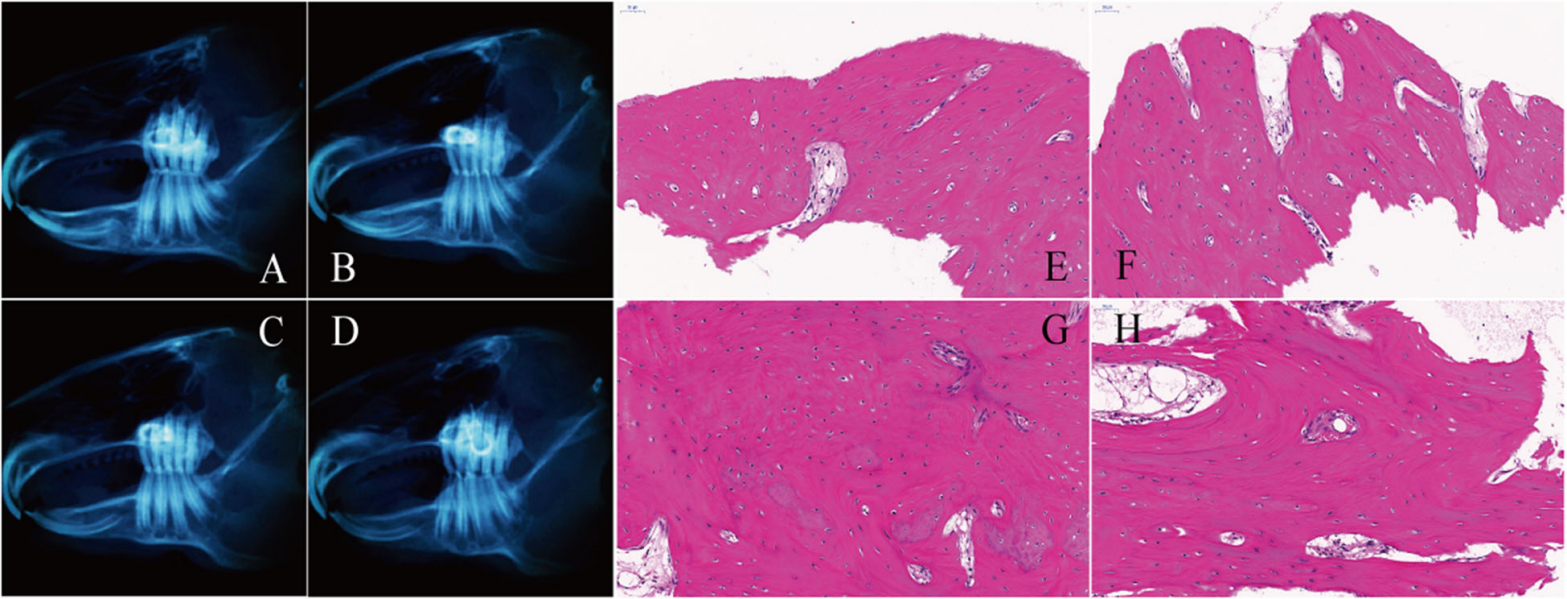
CBCT results and histological analysis at the 8th week after surgery. **a**–**d** The CBCT results of the intact group, NGF group, GS group, and the blank group were shown. **e**–**h** Histological analysis of the intact group, NGF group, GS group, and the blank group was detected

The combined results of macroscopic observation, CBCT examination, and histological analysis showed that the fractures in the NGF group, intact group, GS group, and blank group had completely healed, the osteotomy line had disappeared, and the degree of callus reconstruction was similar and tended to normal bone tissue structure (Fig. 6a–d). There was no significant difference in the visual observation of neurovascular bundles in each group (Fig. 6e–h).

### Quantitative real-time reverse transcription-polymerase chain reaction

**Fig. 7:**
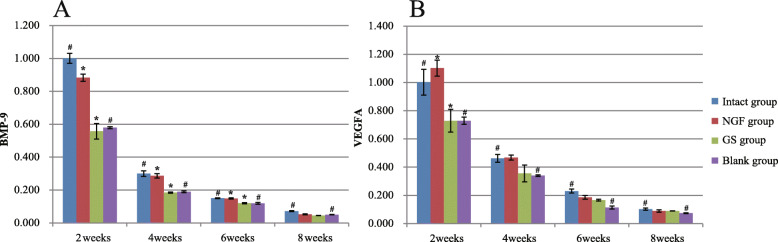
The expression level of BMP-9 mRNA and VEGF mRNA in callus tissues at four stages. **a** The expression levels of BMP-9 mRNA in the intact group, NGF group, GS group, and blank group at the 2nd, 4th, 6th, and 8th weeks were evaluated. **b** The expression levels of VEGF mRNA in the intact group, NGF group, GS group, and blank group at the 2nd, 4th, 6th, and 8th weeks were detected. Asterisk represents a significant difference between NGF group and GS group, *P* < 0.05; number sign represents a significant difference between the intact group and the blank group, *P* < 0.05

At the 2nd, 4th, 6th, and 8th weeks, the expression levels of BMP-9 mRNA and VEGF mRNA in the four groups were detected, respectively. The results showed that the expression levels of BMP-9 mRNA and VEGF mRNA in the four groups reached the highest value at the 2nd weeks, and then decreased with time (Fig. 7, Table S1-S2). At these four time points, the expression levels of BMP-9 mRNA and VEGF mRNA in the intact group were significantly higher than those in the blank group (*P* < 0.05). The expression level of BMP-9 mRNA in the NGF group was significantly higher than that in the GS group except for the 8th week (*P* < 0.05) (Fig. 7a, Table S1). At the 2nd week, the expression of VEGF mRNA in the NGF group was markedly higher than that in the GS group (*P* < 0.05), but there was no statistically significant difference at the other three time points (Fig. 7b, Table S2).

## Discussion

Fracture healing, especially in the mandible, is influenced by a combination of neuromodulation and humoral regulation due to the specific physiological and facial parts [35]. Chisalita et al. found that nerve growth factor (NGF) and insulin-like growth factor 1 (IGF1) played an important role in accelerating the rate of bone healing in patients with brain injury combined with a fracture [11]. In this study, rabbit models of incomplete mandible fracture with mental neurovascular bundle were established in the NGF group, GS group, and blank group, respectively. At the 2nd and 4th weeks after the operation, needling reaction in the lower lip showed the number of animals with nerve reflex recovery was significantly higher in the NGF group than that in the GS group and blank group, and the number of animals with nerve reflex recovery was the same in each group at the 6th and 8th weeks, implying that NGF contributed to early nerve regeneration (Table 2). This result was consistent with the report that NGF was widely distributed in the central and peripheral nervous systems and regulated growth, development, differentiation, regeneration, and functional protein expression of neurons [36]. Chen et al. revealed that after traumatic brain injury combined with fracture, NGF could promote local regulation of nerves and body fluids, and further lead to the involvement of multiple cytokines and nerve regeneration in the bone healing process [7]. Besides, the osteotomy line at the fracture area in the NGF group gradually fused and calcified, and the fracture line disappeared, while the situation in the GS group and blank group was delayed correspondingly at the same times after the operation, suggesting that NGF promoted healing of mandible fracture (Figs. 3, 4, and 5). The result concluded that the serum levels of NGF content were higher in the fracture combined with the brain injury group than in the control group [36, 37]. This result was also verified by the report that NGF stimulated osteoblasts to promote bone cell growth by phosphorylation of NGF receptors [38]. Therefore, NGF, as an important bridge transmitter, could affect the metabolism of bone tissues in the nervous system and then play an important role in the healing of mandible fractures.

NGF promotes fracture healing as follows: inducing nerve growth in bone callus, interaction with neuropeptide substance, regulating bone growth factors, promoting angiogenesis factors in callus, and inhibiting osteoclast function [10]. In this study, there was obvious adhesion at the broken ends of angiogenesis in the NGF group than in the GS group at the 2nd and 4th weeks (Figs. 3b and 4b). Macroscopic observation and CBCT examination showed that a large number of some vascular endothelial cells were found around the trabecular bone. Eppley et al. initially reported that through the application of NGF in mandibular defect repairs in experimental rabbits, NGF not only promoted axonal regeneration but also found that newborn osteoid was generated around the regenerated axons [17]. NGF has been demonstrated to stimulate axonal growth of sensory nerves as well as sympathetic fibers [18] and to promote the expression of vascular endothelial growth factor in the fracture healing process [10]. Our results also suggested that NGF would facilitate angiogenesis and axonal growth at the fracture site during the early stage of fracture healing (Fig. 3f). Besides, CBCT examination revealed a better amount of callus formation and reconstruction, and a more uniform distribution of collagen in the bone matrix depending on the effect of exogenous NGF, implying that NGF could promote the proliferation and differentiation of bone cells to reconstruct the mandible (Figs. 3b, 4b, and 5b). Zhuang et al. showed that a high concentration of NGF could promote the growth of bone callus in fractures and improve the rate of fracture repair, which is consistent with the results of this study [12]. Our findings concluded that NGF could facilitate angiogenesis and the growth of bone callus to promote fracture repair.

As proteins are secreted by cells, growth factors act on critical functions like cell division, matrix synthesis, and tissue differentiation [34, 39], and play an important role in the healing of bone fractures [40]. These bone growth factors include bone morphogenetic protein, vascular endothelial growth factor, fibroblast growth factor, transforming growth factor-β, and so on. Among them, BMP-9 and VEGF are responsible for osteogenesis and angiogenesis, respectively [41–44]. In this paper, a large number of osteoblasts and some vascular endothelial cells were found around the trabecular bone in the NGF group at the 2nd week after the operation (Fig. 3f), while the expression levels of BMP-9 mRNA and VEGF mRNA in the four groups reached the highest value, and the content of BMP-9 and VEGF in the callus tissue of the mandibular fracture area was significantly increased in the NGF group than in the GS group at the same time (*P* < 0.05) (Fig. 7). Hayami et al. indicated that VEGF induced the ingrowth of vessels in cartilage, leading to endochondral bone augmentation, which is very favorable for fracture healing [45]. Song et al. reported that BMP-2 used alone could induce a surplus of callus formation (heterotopic ossification) [46]. After a fracture, systemic or local regulation of nerves and body fluids presented fracture localization, and multiple cytokines and nerve regulation participated in the process of bone healing, especially BMP and VEGF. Lv et al. reported a study to observe the effect of NGF on the expression of BMP-2 in rabbit models [10]. Forriol et al. found that the combination of bone allograft with platelet-rich plasma, rhBMP-7 demonstrated osteoconductive as well as osteoinductive properties for the reconstruction of bony mandibular defects in sheep [47]. The local application of NGF promoted BMP-2 peaked in 2 weeks and decreased later with time, which built the foundation for our study. Comparing with the use of BMP-2 alone, our study found that NGF stimulated a simultaneous increase in the concentration of growth factors such as BMP-9 and VEGF, leading to binding to the receptors on the cell surface of the fracture or damaged site and accelerating the process of fracture repair.

This study clarified the effects of exogenous nerve growth factor on the expression of BMP-9 and VEGF in the early stage of mandible fracture healing in rabbits. In detail, the acupuncture reaction indicated that the number of animals with nerve reflex recovery was higher in the NGF group at the 2nd and 4th weeks after the operation, and then remained consistent in the four groups. Similar manifestations were observed in the radiographic and histological appearance, in which the osteotomy line in the fracture area became blurred in the NGF group, the amount of callus formation and the degree of fusion were better, the process of callus reconstruction remained obvious, and a large number of osteoblasts were still visible around the trabecular bone at the 2nd and 4th weeks. And then, there was no significant difference in acupuncture reaction, CBCT, and histological results among the groups at the 8th week. QRT-PCR showed that the expression of VEGF and BMP-9 mRNA in the NGF group was markedly higher than that in the GS group at the 2nd week (*P* < 0.05), but there was no significant difference at the 4th, 6th, and 8th weeks. The above results concluded that the exogenous NGF facilitated the healing rate at early stage of mandible fracture.

## Limitations

It has been reported that NGF could promote the proliferation and differentiation of bone cells and inhibit the formation of osteoclasts through endocrine factors, such as other neuropeptides and hormones, thereby promoting fracture healing [24]. However, the promotion of fracture healing by NGF might be achieved by regulating a variety of bone growth factors instead of BMP-9 and VEGF only. NGF promotes fracture healing via inducing nerve growth in bone callus, interaction with neuropeptide substance, regulating bone growth factors, promoting angiogenesis factors in callus, and inhibiting osteoclast function [10]. The present study only evaluated the effect of BMP-9 and VEGF on the healing of rabbit mandible fracture with local nerve injury and other BMP families, such as BMP-2 [10] and BMP-7 [47], may play the same role as BMP-9, which lacked identification in this study. Besides, the experiment to verify the function of BMP-9 and VEGF on mandibular fracture healing in rabbits by QRT-PCR was somewhat weak. Therefore, the mechanism of NGF promoting the expression of related neuropeptides in peripheral nerves and affecting the healing of mandibular fractures needs to be further investigated. In the future, we will experimentally investigate the regulating effects of NGF on other cytokines and the mechanisms of interaction among the various factors.

## Conclusions

The results of this study demonstrated that the exogenous NGF would promote nerve repair and angiogenesis at the fracture site in the early phase of mandibular fracture and improve the expression of BMP-9 and VEGF in the early phase of mandibular fracture to accelerate the healing of the mandibular fracture.

## Supplementary Information

**Additional file 1: Table S1.** The expression level of BMP-9 mRNA in the callus tissues at four stages.

**Additional file 2: Table S2.** The expression level of VEGF mRNA in the callus tissues at four stages.

## Data Availability

The datasets used and/or analyzed during the current study are available from the corresponding author on reasonable request.

## Abbreviations

BMP-9: Bone morphogenic protein-9
QRT-PCR: Quantitative real-time PCR
NGF: Nerve growth factor
VEGF: Vascular endothelial growth factor
GS: Gelatin sponge
GDF2: Growth differentiation factor 2
CBCT: Cone-beam computed tomography systems
GAPDH: Glyceraldehyde-3-phosphate dehydrogenase
HE staining: Hematoxylin and eosin staining
TGF-β: Transforming growth factor-β
IGF1: Insulin-like growth factor 1
