# Supplementary material for: Effects of exogenous nerve growth factor on the expression of BMP-9 and VEGF in the healing of rabbit mandible fracture with local nerve injury
Source: J Orthop Surg Res. 2021 Jan 21;16:74. doi: 10.1186/s13018-021-02220-z (PMC7818757; doi:10.1186/s13018-021-02220-z)
Supplement: Supplementary file 1 — Additional file 1: Table S1. The expression level of BMP-9 mRNA in the callus tissues at four stages. [file 13018_2021_2220_MOESM1_ESM.docx]

Table S1. The expression level of BMP-9 mRNA in the callus tissues at four stages.

| Group | 2 weeks | 4 weeks | 6 weeks | 8 weeks |
| --- | --- | --- | --- | --- |
| intact | 1.000 ± 0.031^#^, *p* = 0.001 | 0.300 ± 0.017^#^, *p* = 0.004 | 0.151 ± 0.003^#^, *p* = 0.006 | 0.072 ± 0.003^#^, *p* = 0.001 |
| NGF | 0.883 ± 0.022^*^, *p* = 0.002 | 0.287 ± 0.013^*^, *p* = 0.003 | 0.149 ± 0.004^*^, *p* = 0.001 | 0.052 ± 0.003, *p* = 0.059 |
| GS | 0.558 ± 0.047^*^, *p* = 0.002 | 0.185 ± 0.004^*^, *p* = 0.003 | 0.120 ± 0.004^*^, *p* = 0.001 | 0.045 ± 0.000, *p* = 0.059 |
| blank | 0.579 ± 0.006^#^, *p* = 0.001 | 0.190 ± 0.005^#^, *p* = 0.004 | 0.120 ± 0.006^#^, *p* = 0.006 | 0.051 ± 0.001^#^, *p* = 0.001 |

Data presented as mean ± standart deviation. ^#^Significant difference between the intact group and blank group, *P* < 0.05; ^*^Significant difference between the NGF group and GS group, *P* < 0.05
