# Supplementary material for: Effects of exogenous nerve growth factor on the expression of BMP-9 and VEGF in the healing of rabbit mandible fracture with local nerve injury
Source: J Orthop Surg Res. 2021 Jan 21;16:74. doi: 10.1186/s13018-021-02220-z (PMC7818757; doi:10.1186/s13018-021-02220-z)
Supplement: Supplementary file 2 — Additional file 2: Table S2. The expression level of VEGF mRNA in the callus tissues at four stages. [file 13018_2021_2220_MOESM2_ESM.docx]

Table S2. The expression level of VEGF mRNA in the callus tissues at four stages.

| Group | 2 weeks | 4 weeks | 6 weeks | 8 weeks |
| --- | --- | --- | --- | --- |
| intact | 1.003 ± 0.092^#^, *p* = 0.028 | 0.463 ± 0.027^#^, *p* = 0.013 | 0.231 ± 0.015^#^, *p* = 0.001 | 0.103 ± 0.008^#^, *p* = 0.013 |
| NGF | 1.103 ± 0.056^*^, *p* = 0.004 | 0.468 ± 0.018, *p* = 0.072 | 0.187 ± 0.014, *p* = 0.108 | 0.089 ± 0.009, *p* = 0.923 |
| GS | 0.729 ± 0.081^*^, *p* = 0.004 | 0.355 ± 0.060, *p* = 0.072 | 0.166 ± 0.006, *p* = 0.108 | 0.089 ± 0.001, *p* = 0.923 |
| blank | 0.729 ± 0.026^#^, *p* = 0.028 | 0.340 ± 0.006^#^, *p* = 0.013 | 0.114 ± 0.010^#^, *p* = 0.001 | 0.074 ± 0.003^#^, *p* = 0.013 |

Data presented as mean ± standart deviation. ^#^Significant difference between intact the group and blank group, *P* < 0.05; ^*^Significant difference between the NGF group and GS group, *P* < 0.05
